# Multi-branch fusion graph neural network based on multi-head attention for childhood seizure detection

**DOI:** 10.3389/fphys.2024.1439607

**Published:** 2024-10-31

**Authors:** Yang Li, Yang Yang, Shangling Song, Hongjun Wang, Mengzhou Sun, Xiaoyun Liang, Penghui Zhao, Baiyang Wang, Na Wang, Qiyue Sun, Zijuan Han

**Affiliations:** ^1^ School of Information Science and Engineering, Shandong University, Qingdao, China; ^2^ Bidding Office, The Second Hospital of Shandong University, Jinan, China; ^3^ Institute of Research and Clinical Innovations, Neusoft Medical Systems Co., Ltd., Beijing, China; ^4^ Institute of Research and Clinical Innovations, Neusoft Medical Systems Co., Ltd., Shanghai, China; ^5^ Center for Optics Research and Engineering, Shandong University, Qingdao, China

**Keywords:** childhood seizure detection, graph convolutional network, adjacency matrix, EEG, multi-head attention

## Abstract

The most common manifestation of neurological disorders in children is the occurrence of epileptic seizures. In this study, we propose a multi-branch graph convolutional network (MGCNA) framework with a multi-head attention mechanism for detecting seizures in children. The MGCNA framework extracts effective and reliable features from high-dimensional data, particularly by exploring the relationships between EEG features and electrodes and considering the spatial and temporal dependencies in epileptic brains. This method incorporates three graph learning approaches to systematically assess the connectivity and synchronization of multi-channel EEG signals. The multi-branch graph convolutional network is employed to dynamically learn temporal correlations and spatial topological structures. Utilizing the multi-head attention mechanism to process multi-branch graph features further enhances the capability to handle local features. Experimental results demonstrate that the MGCNA exhibits superior performance on patient-specific and patient-independent experiments. Our end-to-end model for automatic detection of epileptic seizures could be employed to assist in clinical decision-making.

## 1 Introduction

Epilepsy is a neurological disorder characterized by abnormal synchronous discharges of neurons. Childhood seizures carry a risk for the presence of cognitive impairment and behavioral disorders (Rennie et al., 2004). Therefore, accurate detection of seizures in children is of paramount importance for determining the best treatment plans and preventing adverse conditions. The diagnosis of epilepsy typically relies on the analysis of electroencephalogram (EEG), which is abnormal in the majority of patients. However, this task requires highly experienced experts who must invest a significant amount of time and effort in inspecting lengthy EEG recordings (Kannathal et al., 2005). This process is also susceptible to the subjective influence of epilepsy experts.The occurrence of epileptic seizures significantly impacts the physical development of children, emphasizing the necessity for early detection and intervention in pediatric epilepsy to mitigate its effects. Therefore, designing a reliable childhood seizure detection model can facilitate the automation of epilepsy diagnosis, holding significant importance in enhancing the quality of life for children.

There is a significant need to expand the utilization of machine learning, particularly within the emerging realm of deep learning, for automating the detection of epilepsy through EEG signal classification. Among the extensively explored techniques for EEG seizure detection, feature extraction rooted in machine learning stands as one of the most prominent approachs. For instance, multiscale entropy were extracted using extreme learning machines (Cui et al., 2017), nonlinear features were extracted using Gradient Boosting Decision Trees (GBDT) (Xu et al., 2022), various features were extracted using Empirical Mode Decomposition (EMD) (Singh et al., 2019), multi-scale features were extracted using wavelet transformation (Zhang et al., 2010), and the Comprehensive Representation of K Nearest Neighbors (CRMKNN) (Na et al., 2021) approach is proposed for epilepsy diagnosis. The traditional machine learning algorithms often struggle to achieve automatic detection of epilepsy, and experimental results are influenced by empirical parameters, making it difficult to stabilize the algorithm’s performance. In essence, time series data is nonlinear and dynamic, making it challenging for traditional machine learning algorithms to effectively capture these complex signal characteristics. Moreover, there are large variations between different patients. Therefore, traditional machine learning faces challenges in learning the hidden features of EEG signals and lacks generalization.

Compared to traditional machine learning, neural networks, as a more promising algorithm with greater capacity for learning from complex data, have been applied to various research fields (Zhao P. et al., 2022; Sun and Yang, 2023; Chen J. et al., 2024; Abu and Diamant, 2023). There have been numerous advancements in the detection of epilepsy EEG signals as well (Wang et al., 2023a; Zhao et al., 2023b; He et al., 2022; Xiao et al., 2024). A novel deep network called Two-Stream 3-D Attention Module (TSA3-D) (Cao et al., 2022) was introduced to leverage the multichannel time-frequency and frequency-space features of interictal EEGs for epilepsy classification. In (Feng et al., 2022) a 3D deep network combined with residual attention modules was proposed to explore the spatial and time-frequency features of multi-channel EEG. In (Cui et al., 2022b), a fusion model based on transfer learning and time-frequency features was proposed for the effective detection of childhood epilepsy syndrome. In (Cui et al., 2022a), an analysis of the correlation between time-frequency features and EEG signals was conducted, and a childhood epilepsy syndrome classification model based on transfer networks was proposed.

Attention mechanisms have gained widespread applications in the field of signal recognition (Zhao et al., 2023b; Lian and Xu, 2023; Peh et al., 2023; Qiu et al., 2023; Wang Z. et al., 2023; Liu et al., 2023), emerging as a pivotal technology attracting significant attention and in-depth exploration within the realm of deep learning. Many researchers have integrated attention mechanisms with neural network models, resulting in the creation of a series of innovative models. The emergence of these models has introduced new possibilities for enhancing the accuracy and efficiency of EEG signal recognition, thus steering the direction of development in this field. Ding et al. (2023) proposed a novel seizure prediction model that utilizes a CNN to automatically capture features from EEG signals. This model combines multiple head attention mechanisms to identify relevant information within these features for the recognition of EEG signal segments. Deng et al. (2023) introduced a novel hybrid vision transformer (HViT) model that could enhance the multi-head attention mechanism by augmenting the capability of convolution to process local features, thereby achieving data uncertainty learning. Zhao et al. (Zhao X. et al., 2022) proposed a recommendation detector based on multi-head attention mechanism, utilized for detecting pathological high-frequency oscillations (HFOs) associated with epilepsy to locate the epileptogenic zones. The attention mechanism aids the network in capturing dependencies among features and enhancing the model’s sensitivity to local information. In the field of seizure detection, the potential of attention mechanism remains to be further explored.

In the epilepsy detection process using deep learning, EEG signals are represented as two-dimensional signals, considering only channel-based features and disregarding information about the physical distribution of channels. The electrode distribution in EEG exhibits a non-Euclidean topological structure, which can lead to the loss of connectivity information between brain functional regions, neglecting the long-term interdependencies among EEG signals from different channels. The graph convolutional network (GCN) algorithms can effectively leverage the implicit graph representation information within EEG signals. GCN algorithms utilize graph structures and update graph representations through node aggregation. GCN has been widely applied in numerous EEG signals, which have demonstrated excellent performance, such as emotion recognition (Song et al., 2021; Liu et al., 2022; Chen Y. et al., 2024; Li Y. et al., 2022), Alzheimer’s disease (Lopez et al., 2023), automatic seizure detection (Wagh and Varatharajah, 2020; Meng et al., 2022; Ho and Armanfard, 2023), driver state monitoring (Kalaganis et al., 2020), motor imagery (Cai et al., 2022), and sleep stage classification (Li M. et al., 2022; Jia et al., 2020; Lee et al., 2024; Ji et al., 2022; Jia et al., 2021). Wang et al. (2022) proposed a spatiotemporal graph attention network (STGAT) based on phase locking value (PLV) to extract spatial and functional connectivity information. Raeisi et al. (2022) constructed graph representations using three different types of spatial information and assessed the performance of neonatal seizure detection. He et al. (2022) utilized the graph attention network (GAT) to extract spatial features and employed a bi-directional long short-term memory network (BiLSTM) to capture temporal relationships before and after the current time frame for epilepsy detection.

The integration of attention mechanisms with GCN has proven effective in enhancing model performance on graph-structured data (Wu et al., 2024; Li et al., 2023; Cheng et al., 2023; Dong et al., 2022; Wang Y. et al., 2023; 2020; Grattarola et al., 2022). Attention helps highlight important nodes or features, improving the ability of GCN to capture both global and local relationships, which is crucial for EEG classification. The dynamic temporal graph convolutional network (DTGCN) (Wu et al., 2024) is proposed for seizure detection and classification, incorporating a seizure attention layer to capture the distribution patterns of epilepsy and a graph structure learning layer to represent the dynamically evolving graph structure in the data. A spatiotemporal hypergraph convolutional network (STHGCN) (Li et al., 2023) is designed to capture higher-order relationships in EEG recordings, construct feature hypergraphs across the spectral, spatial, and temporal domains to focus on EEG channel correlations and dynamic temporal relationships, and integrate self-attention mechanisms to initialize and update relationships within EEG sequences. A hybrid network (Cheng et al., 2023) is proposed, consisting of a Dynamic Graph Convolution (DGC) module and a Temporal Self-Attention Representation (TSAR) module. This network simultaneously integrates representative knowledge of spatial topology and temporal context into the EEG emotion recognition task. In summary, the integration of attention mechanisms with GCN allows for more effective feature representation by dynamically weighting the importance of spatial and temporal relationships, thereby enhancing the ability of model to capture complex dependencies in EEG data for emotion recognition tasks.

During the training process, traditional GCN describes the dynamic process of epileptic seizures using a single graph representation. During seizures, various complex interactions of neural activities occur, including different types of brainwave changes and alterations in connectivity patterns between brain regions. Therefore, the dynamic process of epileptic seizures cannot be exhaustively represented by a single static graph. To comprehend and describe seizures, it is necessary to consider the changes in the temporal dimension, and the interactions between different brain regions in the spatial dimension. Therefore, this study introduces a multi-branch graph convolutional model with multi-head attention (MGCNA) for childhood seizure detection. Specifically, the MGCNA employs three graph representation approaches to characterize the feature representation of EEG data from multiple dimensions. By incorporating a multi-head attention mechanism, it combines spatial topological information from multi-channel electrodes with dynamic temporal information, enhancing the global contextual awareness and recognition capabilities of model. The major contributions of MGCNA can be summarized as follows:

• A multi-branch GCN model is proposed. It utilizes Euclidean distance to capture spatial information between channels, employs Pearson correlation coefficient to gather functional connectivity information among channels, and supplements latent spatiotemporal correlations through a trainable adjacency matrix. The multi-channel EEG signals are modeled as graph signals, enabling the extraction of synchrony relationships within EEG signals.
• By integrating graph signals with a multi-head attention mechanism, attention weights for graph features are obtained, and the hidden vector representation of graph signals is derived through the summation of weighted values.
• We conduct patient-specific and patient-independent experiments to assist doctors in rapidly identifying the onset period in complex scenarios. The outcomes from these two experiments showcase the effectiveness of our method. Through comparison with other methods, the sensitivity, which is of utmost clinical concern, is the highest in two experiments.

The organization of the rest of this article is as follows. In Section 2, the proposed seizure detection model is introduced in terms of the overall MGCNA structure, extraction of intrachannels features, multi-branch GCN, multi-head attention mechanism, and classifier module. Section 3 presents the implementation details, performance evaluation metrics and experimental results of the CHB-MIT dataset. Section 4 interprets the results and emphasizes the limitations of the current study, and the conclusion is provided in Section 5.

## 2 Methods

In this section, we introduce our proposed MGCNA model for epilepsy classification using multi-channel EEG signal.

### 2.1 General structure

**FIGURE 1:**
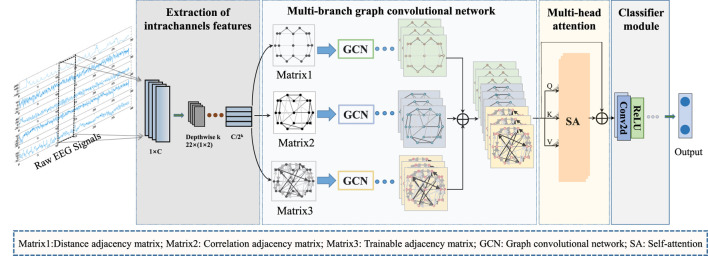
The overall architecture of MGCNA. There are mainly four steps including extraction of intrachannels features module; Multi-branch GCN; Multi-head attention module and Classifier module.

Figure 1 provides an overview of the overall process for classifying epileptic EEG signals using the MGCNA proposed in this article. First, intrachannels features are extracted from the raw EEG signals to create graph signals. Second, three different time-series-to-graph representation are employed and graph features are extracted through GCN. Third, a multi-head attention is utilized to learn dependencies of different graph features. Finally, the combined graph features are processed to output classification results.

### 2.2 Extraction of intrachannels features

Convolution has been proven to be highly effective in capturing features of EEG signals. Inspired by the EEGWaveNet (Thuwajit et al., 2021) model, this module utilizes depthwise convolution to compress the input signals’ resolution in a channel-wise manner and capture features. Depthwise convolution does not cross information between channels and extracts features independently. This module comprises k consecutive layers, where each layer captures valuable features within channels at half the scale of the resolution of the previous layer.

In this module, the input data X are shaped as (n,1,C) , where n and C represent the number of channels and the number of sampling points, respectively. n is set to 22, and C is defined as f×t , where f denotes the EEG’s sampling frequency, and t is the duration of each epoch’s segmentation. In each layer, depthwise convolution is applied to each channel with a kernel size of (1, 2), a stride of 2, and no padding. The output size of each k th layer is ( n , 1, $C/2^{k}$ ).

**FIGURE 2:**
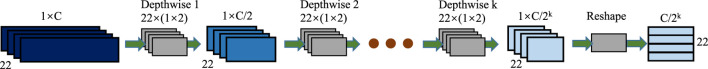
The data flow for extracting intrachannels features.

The module of extraction of intrachannels features can be represented as:

$$F^{k}=DWConv\left(F^{k-1}\right)$$ (1)

where $F^{0}$ is the input signal $X\in R^{n\times 1\times C}$ , DWConv(⋅) is the depthwise convolution. After reshape, the features extracted by this module $N\in R^{n\times \left(C/2K\right)}$ will be fed into the next module to obtain multi-scale graph features. Figure 2 depicts the process of extracting intrachannels features.

### 2.3 Multi-branch graph convolutional network

**FIGURE 3:**
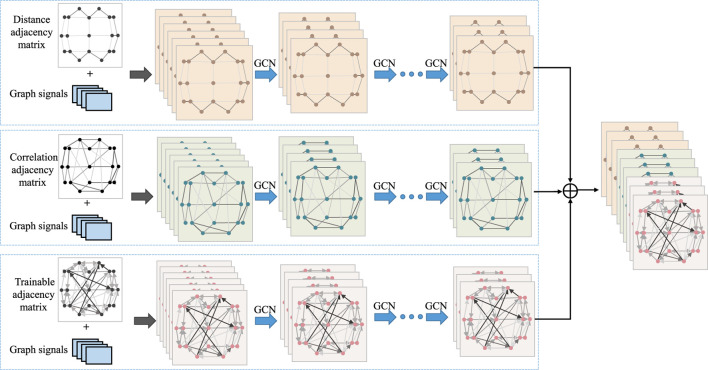
The pipeline of multi-branch graph convolutional network.

To design an appropriate graph representation for EEG signals, it is necessary to construct a graph based on the physical distribution of channels and the spectral-temporal domain characteristics of the signals. Each channel was considered as a node and the connection of each node is characterized in terms of spatial position information, EEG signal similarity features, and a learnable graph representation to dynamically capture the topological relationship between EEG signal channels. These three types of graph signals are input into GCN, allowing us to leverage the structural characteristics of EEG signals to capture spatial dependencies between nodes. Figure 3 illustrates the overall process of multi-branch GCN. The graph convolution processes at the top, middle, and bottom are based on spatial distance graph representation, functional connectivity graph representation, and adaptive graph representation, respectively.

#### 2.3.1 Graph generator

Graph representation consists of graph signal and graph. Graph signals are extracted from extraction of intrachannels features module. A graph is denoted as G={V,ϵ,A} , where V is the set of nodes, ϵ is the set of edges, and A is the adjacency matrix. Graph nodes represent EEG channels. The connections between nodes are controlled by the adjacency matrix A . To construct the adjacency matrix A , this paper employs three graph learning methods: information based on spatial distance between nodes, measure based on functional connectivity, and adaptive graph learning methods.

##### 2.3.1.1 Spatial distance graph learning

Seizures are produced by abnormal nerve discharges in different areas of the cerebral cortex and cause significant changes in the EEG. And this abnormal discharge spreads and affects neighboring brain regions, making strong correlation between adjacent areas. So the spatial distance between nodes of EEG signals is used for graph representation in EEG signal analysis.

In this approach, we consider the electrode positions of the EEG as a three-dimensional grid model. To represent the spatial distance of EEG electrodes, we compute adjacency matrix $A1_{ij}$ by applying Euclidean distance between pairs of channels in the bipolar montage. The distance between pairs of channels $v_{i}\left(x_{i},y_{i}\right)$ and $v_{j}\left(x_{j},y_{j}\right)$ , which can be denoted as:

$$Dist_{ij}=sqrt\left({\left(x_{i}-x_{j}\right)}^{2}+{\left(y_{i}-y_{j}\right)}^{2}\right)$$ (2)

where $v_{i}\left(x_{i},y_{i}\right)$ and $v_{j}\left(x_{j},y_{j}\right)$ are the centers of the bipolar derivations. The spatial distances obtained from Euclidean distance are transformed into the adjacency matrix while taking into account the strength of connections between nodes. To calculate the adjacency matrix, we apply the following rules:

$$\begin{array}{c} A1_{ij}=\left\{\begin{array}{cc} exp\left(-\frac{Dist_{ij}^{2}}{\delta^{2}}\right),\; & ifDist_{ij}\leq R_{1}\\ 0,\; & otherwise \end{array}\right. \end{array}$$ (3)

where $R_{1}$ is the threshold for sparsity and δ is the standard deviation of the distances. This results in a universally undirected, weighted graph for all EEG graph representation.

##### 2.3.1.2 Functional connectivity graph learning

The strength of association between channels varies during seizures. and the synchronization between brain regions can be assessed by calculating the connectivity between channels. Therefore, we investigate the relationship of information interaction between channels during seizures.

The functional connectivity between nodes of EEG signals is used for graph representation in EEG signal analysis. To represent the functional connectivity of EEG electrodes, we compute adjacency matrix $A2_{ij}$ by applying Pearson correlation coefficient between the preprocessed signals in $v_{i}$ and $v_{j}$ . The Pearson correlation coefficient between pairs of channels $v_{i}$ and $v_{j}$ , which can be denoted as:

$$\rho_{ij}=\frac{\sum \left(X_{i}-\bar{X_{i}}\right)\left(X_{j}-\bar{X_{j}}\right)}{\sqrt{\sum {\left(X_{i}-\bar{X_{i}}\right)}^{2}\sum {\left(X_{j}-\bar{X_{j}}\right)}^{2}}}$$ (4)

where $X_{i}$ and $X_{j}$ are graph signals extracted from extraction of intrachannels features module in $v_{i}$ and $v_{j}$ . The computed correlation coefficients are normalized to the range [0, 1]. The functional connectivity obtained from Pearson correlation coefficient are transformed into the adjacency matrix while taking into account the strength of connections between nodes. To calculate the adjacency matrix, we apply the following rules:

$$\begin{array}{c} A2_{ij}=\left\{\begin{array}{cc} \rho_{ij},\; & if\rho_{ij}\geq R_{2}\\ 0,\; & otherwise \end{array}\right. \end{array}$$ (5)

where $R_{2}$ is the threshold. This results in an undirected, weighted graph for all EEG graph representation.

##### 2.3.1.3 Adaptive graph learning

The human brain possesses extremely complex structure and functionality, and analyzing it merely through spatial distance and functional connectivity graph structures does not fully capture its functions and behavioral manifestations. The neural network model aims to simulate the interconnection and information transfer between neurons in the human brain, enabling learning and reasoning of complex tasks. Therefore, we incorporate graph structures as part of the parameters in neural networks, training them to capture the coupling relationships with EEG signals.

The adaptive graph learning method can learn the intrinsic connections between EEG signals. In the adaptive graph learning method we employed, the adjacency matrix $A3\in R^{n\times n}$ , which characterizes the relationships between individual vertex nodes, is dynamically learned rather than predetermined. During the model training process, the adjacency matrix A3 of the adaptive graph representation is updated during training through back propagation.During the training process, $A_{3}$ will be constrained by the following formula:

$$A3^{l}=ReLU\left(BN\left(a3^{l}\right)\right)$$ (6)

where $a3^{l}$ is the adjacency matrix obtained in the l -th layer, using BN and ReLU to prevent overfitting and improve stability. Due to the fact that the closer spatial connection between channels does not accurately reflect a closer functional relationship between them, and functional connectivity does not fully characterize the intrinsic relationship between channels (Song et al., 2018), we utilize the adaptive graph learning method to capture the inherent connections among EEG channels and enhance EEG recognition.

#### 2.3.2 Graph convolution

A general GCN model (Kipf and Welling, 2016; Defferrard et al., 2016) takes graph signals $N\in R^{n\times \left(C/2K\right)}$ and adjacency matrices $A\in R^{n\times n}$ as inputs and a graph feature matrix $Z\in R^{M\times P}$ is generated through node aggregation. P represents the dimension of the output feature vector for each node. GCN updates node features by aggregating features from neighboring nodes. The graph convolutional layer can be represented as follows:

$$H^{l}=ReLU\left({\tilde{D}}^{-\frac{1}{2}}\tilde{A}{\tilde{D}}^{-\frac{1}{2}}H^{l-1}\theta^{l}\right)$$ (7)

where $\tilde{A}$ represents adjacency matrix containing diagonal matrix. $\tilde{D}$ denotes the degree matrix for each node in the graph. $H^{l-1}$ represents the graph feature matrix of the previous output, and $H^{0}\in R^{n\times \left(C/2K\right)}$ is the graph signal N . $\theta^{l}$ represents the matrix of learnable parameters.

Notably, the structures of the three GCN branches are identical, but their parameters are not shared. After each GCN, Batch Normalization (BN) is applied to normalize the graph features at each layer, which helps improve the generalization capability of network. And it is presented as the following form:

$$Z_{i}^{l}=BN\left(H_{i}^{l}\right)$$ (8)

where $z_{i}^{l}$ is the input of the next graph convolution layer. i indicated as the i th branch of the GCN. The final output of multi-branch GCN is expressed as the following formula:

$$Z=\left[z_{1}^{m},z_{2}^{m},z_{3}^{m}\right]$$ (9)

where m represents the number of layers of the GCN, and [⋅] represents concatenation.

### 2.4 Multi-head attention mechanism

The attention mechanism, which is a deep learning model, emulates the pivotal information and critical elements that individuals concentrate on during observations. The multi-head attention mechanism makes the output of attention to incorporate encoding from different spatial locations, thereby enhancing the expressive power of the model. In standard multi-head attention with input matrix $Z\in R^{m\times n\times D}$ , the self-attention for each head are computed according to the following formula:

$$Head\left(Q,K,V\right)=Softmax\left(\frac{QK^{T}}{\sqrt{d}}\right)V$$ (10)

where Q , K , V represent the query vector, key vector and value vector, respectively. h is the number of heads, d=m/h . The original multi-head attention mechanism has a high computational cost, with a quadratic complexity of $O\left(L^{2}d\right)$ , where L is n×D .

**FIGURE 4:**
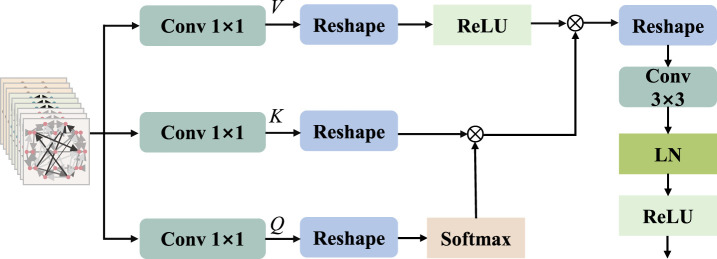
The process of self-attention.

Inspired by MobileViT v2 (Mehta and Rastegari, 2022) and HViT-DUL (Deng et al., 2023), the multi-head design is incorporated into the separable self-attention mechanism, which is applied to the proposed model for the reduction of computational overhead. The separable multi-head attention mechanism employed in this paper has a linear complexity of O(L) , which is lower compared to the standard multi-head attention mechanism. When multiple self-attention mechanisms are sequentially linked and subsequently subjected to a final projection, it results in the generation of the ultimate values for the multi-head attention mechanism. The spatial topological information and dynamic variation information of multi-channel electrodes are obtained through a multi-head attention mechanism to combine representations of multiple graph signals, thereby enhancing the discrimination ability. Figure 4 illustrates the process of separable multi-head attention. ***Q***, ***K***, ***V*** is calculated using the following formula:

$$\begin{array}{c} \left\{\begin{array}{c} Q=Conv_{1}\left(Z\right)\;\\ K=Conv_{2}\left(Z\right)\;\\ V=Conv_{3}\left(Z\right)\; \end{array}\right. \end{array}$$ (11)

where Z is the output of multi-branch GCN features. Conv is two-dimensional convolution. We employ three 1×1 convolutional layers, with parameters not shared, to compute the QKV of the input graph features. After the reshaping process, the outputs Q , K , V are in the following dimensions: $Q\in R^{h\times 1\times n\times D}$ , $K\in R^{h\times d\times n\times D}$ and $V\in R^{h\times d\times n\times D}$ .

To calculate self attention scores, we apply the Softmax operation to the Q and taking the element-wise multiplication with K , After that, we perform element-wise multiplication with V after passing it through ReLU. Self attention scores $Atten_{i}$ are computed based on the following formula:

$$atten_{i}=ReLU\left(V\right)*\left(K*SoftmaxQ\right)$$ (12)

where atten is the self-attention scores. ReLU is activation function. After concatenating the scores of multiple self-attention mechanisms, we perform a 3×3 convolution on the resulting features, normalize it using BN, and then apply ReLU to enhance generalization ability. The computed multi-head attention scores, matching the dimensions of the input Z , are added to the output of multi-branch GCN, expressed as:

$$Atten=\left[atten_{1},atten_{2},\ldots ,atten_{h}\right]$$ (13)

$$MA=ReLU\left(BN\left(Conv\left(Atten\right)\right)\right)$$ (14)

$$Z^{\prime }=BN\left(Z+MA\right)$$ (15)

where [⋅] represents the concatenation.

### 2.5 Classifier module

**FIGURE 5:**
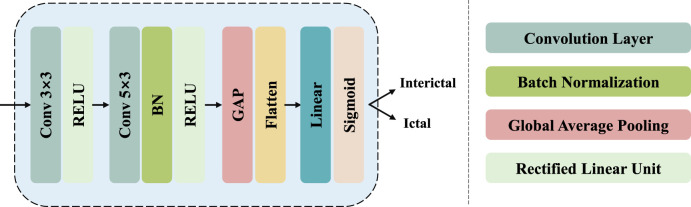
The structure of classifier module.

A classifier module is placed after the multi-head attention mechanism for the final class inference. Initially, it involves two layers of 2D convolution, with a ReLU activation function applied after each convolution to enhance the robustness of model. This process can be described as:

$$F^{\prime }=ReLU\left(Conv\left(ReLU\left(Conv\left(Z^{\prime }\right)\right)\right)\right)$$ (16)

where $Z^{\prime }$ is the output of multi-head attention mechanism. Global average pooling pools each channel of the convolution result, followed by a linear layer that reduces the feature dimensionality to match the number of classes. After passing through a sigmoid operation, the index with the highest probability is selected as the classification result. This process can be described as:

$$Pre^{s}=\sigma \left(Linear\left(AvgPool\left(F^{\prime }\right)\right)\right)$$ (17)

where AvgPool(⋅) denotes global average pooling, σ is sigmoid operation, Linear(⋅) is the Linear operation, and $Pre^{s}$ is the final classification result. Figure 5 illustrates the structure of the classifier module.

### 2.6 Training procedure

In order to achieve optimal network parameters during the training process, we employ the backpropagation (BP) algorithm, which iteratively updates the network parameters until an optimal or suboptimal solution is reached. In this context, we introduce a loss function based on binary cross entropy, defined as follows:

$$loss=cross\text{\_}entropy\left(Pre,Pre^{s}\right)+\tau \|\Omega \|$$ (18)

$$cross\text{\_}entropy\left(Pre,Pre^{s}\right)=-\left[Pre\log Pre^{S}+\left(1-Pre\right)\log\left(1-Pre^{S}\right)\right]$$ (19)

where Pre and $Pre^{s}$ represent the actual label and the predicted label, respectively. The binary cross entropy, denoted as $cross\text{\_}entropy\left(Pre,Pre^{s}\right)$ , quantifies the disparity between the true labels and the predicted labels. τ represents the trade-off parameter, while ‖⋅‖ denotes the l 2-norm to prevent overfitting. Ω represents all the parameters within this model.

For adaptive graph learning, the adjacency matrix A3 is a trainable parameter within the network that optimizes with model optimization. The partial derivatives of the loss function with respect to the optimal adjacency matrix A3 and the loss are expressed as follows:

$$\frac{\partial loss}{\partial A}=\frac{cross\text{\_}entropy\left(Pre,Pre^{s}\right)}{\partial A}+\tau \frac{\left\|\Omega \right\|}{\partial A}$$ (20)

### 2.7 Dataset

The CHB-MIT dataset (Shoeb, 2009) was collected by Boston Children’s Hospital and is currently the most widely used public dataset. The multichannel scalp EEG signals consists of EEG signal recordings from 23 children with epilepsy at a sampling rate of 256 Hz. The EEG signals are collected using EEG electrodes placed according to the International 10–20 system. The dataset comprises approximately 983 h of EEG recordings, including 198 seizure onset events, with a total duration of 3 h 15 min.

### 2.8 Implementation details

In the CHB-MIT dataset, most records have 23 channels, while some records have missing or duplicated channels. In this study, 22 channels are selected in this study to maintain consistency of channels among all patients. The selected 22 channels are FP1-F7, F7-T7, T7-P7, P7-O1, FP1-F3, F3-C3, C3-P3, P3-O1, FP2-F4, F4-C4, C4-P4, P4-O2, FP2-F8, F8-T8, T8-P8, P8-O2, FZ-CZ, CZ-PZ, P7-T7, T7-FT9, FT9-FT10, FT10-T8. This dataset is classed into two categories, including interictal and ictal. To reduce the effect of noise, a fifth-order Butterworth band-pass filter ranging from 0.5 Hz to 70 Hz was used. The filtered EEG signals are segmented into 3-s windows. Due to the scarcity of ictal EEG data compared to interictal EEG data, the windows overlap by 50 % to obtain more ictal data.

For a comprehensive evaluation of the MGCNA, we train both a patient-independent model and a patient-specific model. In a patient-specific approach, the model is trained, validated, and tested using data from an individual subject. Interictal signals are randomly discarded for each subject, and the ratio of interictal signals to ictal signals is maintained at 5:1. As the number of ictal signals is significantly lower than that of interictal signals, which is detrimental to model training, we employ re-sampling to create a balanced training dataset. In each epoch of training, a random selection of an equal number of interictal signals is made to match the ictal signals in the training set, followed by random shuffling. Consequently, we obtain a balanced training subset for each epoch. We employ a 5-fold cross-validation approach and report the average performance. In a patient-independent approach, the model was trained and validated using data from multiple subjects, and then tested on data from individual subject. We utilized the leave-one-subject-out cross-validation as evaluation method. The data in both categories are kept balanced. The patient-specific approach emphasizes individual differences and personalization, and the patient-independent approach focuses more on overall trends and general patterns.

We employ the Adam optimizer with a learning rate of 3e-4, a weight decay of 1e-3.The dropout rate is experimentally set to 0.5. $R_{1}$ is set to 0.4 and $R_{2}$ is set to 0.25. k is set to 2. All the above experiments were performed and implemented by Pytorch 1.7.1 in the NVIDIA GTX3090 and CUDA11.0 environment.

### 2.9 Performance evaluation metrics

We use eight different performance metrics to evaluate the performance of MGCNA including Accuracy (Acc), Sensitivity (Sen), Specificity (Spe), F1 Score (F1) , and Area Under Curve (AUC). These metrics are obtained using true positives (TP), true negatives (TN), false positives (FP), and false negatives (FN) values.

Accuracy represents the proportion of samples correctly classified by the model out of the total sample count. Sensitivity and Specificity measure the model’s ability to classify positive and negative samples. F1 Score combines precision and recall to provide a comprehensive performance measure. AUC is based on the area under the ROC curve and provides a comprehensive performance metric for unbalanced category distributions.

## 3 Experimental results

### 3.1 Patient-specific experiments

Patient-specific experimental results on CHB-MIT dataset using the MGCNA are shown in Table 1. According to the table, the model demonstrates an average accuracy of 99.32 % , specificity of 98.4 % , sensitivity of 98.74 % , an F1 score of 97.76 % , and an AUC of 98.51 % . For the majority of patients, the recognition accuracy exceeds 97 % , with one patient achieving 100 % accuracy. The model achieves a specificity of 99 % for 12 patients, which represents 50 % of all test subjects, and a sensitivity of 99 % for 17 patients, covering 70 % of all test subjects. F1 scores for all patients are greater than 92 % , and the AUC is above 95 % . These results indicate the stability and high performance of MGCNA proposed in this study, which can aid medical professionals in diagnosis.

**TABLE 1:** Patient-specific experimental results on CHB-MIT dataset using the proposed architecture.

Case	Acc (%)	Sens (%)	Spec (%)	F1 (%)	AUC (%)
CHB01	99.79	99.12	100.00	99.33	99.52
CHB02	99.56	96.96	100.00	98.01	98.44
CHB03	99.75	99.74	99.74	99.20	99.74
CHB04	99.32	96.58	99.50	94.81	98.05
CHB05	99.48	97.40	99.90	98.34	98.65
CHB06	99.94	100.00	99.94	98.84	98.63
CHB07	99.79	100.00	98.82	99.39	99.42
CHB08	99.62	99.82	98.57	98.83	99.20
CHB09	100.00	100.00	100.00	100.00	100.00
CHB10	99.96	100.00	99.74	99.87	99.87
CHB11	99.83	99.92	99.39	99.51	99.66
CHB12	98.63	99.71	93.77	95.67	96.74
CHB13	97.88	99.26	93.51	95.07	96.38
CHB14	98.11	99.07	93.45	94.18	96.26
CHB15	98.31	98.66	97.91	98.21	98.29
CHB16	99.61	100.00	97.50	98.67	98.75
CHB17	99.29	99.79	96.90	97.82	98.34
CHB18	99.55	99.80	98.28	98.60	99.04
CHB19	99.78	100.00	98.57	99.26	99.29
CHB20	98.91	93.61	99.50	94.32	96.56
CHB21	97.98	92.68	98.82	92.81	95.75
CHB22	99.43	99.53	99.41	98.15	99.47
CHB23	99.58	99.80	98.55	98.78	99.18
CHB24	99.48	98.20	99.77	98.57	98.99
Mean	99.32	98.74	98.40	97.76	98.51

### 3.2 Patient-independent experiments

In contrast to the patient-specific experiments, patient-independent experiments involve training a general model for all patients. The experiment employed the leave-one-out method. Patient-independent experiments separate one patient’s signal as a test set, using the EEG data of other patients as the training set and Validation set. This allows the model to detect epileptic activity in the test set by learning common seizure characteristics from the training set. This experiment is more clinically meaningful but requires to address differences in EEG signals among different patients, which may result from physiological variations, equipment noise, etc. Compared to patient-specific experiments, patient-independent experiments make it more challenging for the algorithm to identify epileptic seizures and lead to poorer detection results.

Patient-independent experimental results on CHB-MIT dataset using the MGCNA are shown in Table 2. As seen in the table, the average values for Accuracy, Sensitivity, Specificity, and F1 are all above 80 % . There is a significant individual variation among different patients. Patients ♯ 4, ♯ 5, ♯ 7, ♯ 9, ♯ 10, ♯ 15, ♯ 17, ♯ 18, ♯ 19, ♯ 22, ♯ 23 achieved accuracy rates exceeding 90 % , whereas patients ♯ 8, ♯ 12, ♯ 13, and ♯ 24 had comparatively lower accuracy rates. Compared to patient-specific experiments, patient-independent experiments exhibited a 13.95 % lower accuracy rate. This is due to the differences in seizures between patients and the presence of different types of symptoms during seizures, which vary in frequency and duration.

**TABLE 2:** Patient-independent experimental results on CHB-MIT dataset using the proposed architecture.

Case	Acc (%)	Sens (%)	Spec (%)	F1 (%)	AUC (%)
CHB01	91.51	83.73	99.28	90.79	91.51
CHB02	82.10	64.20	100.00	78.20	82.10
CHB03	81.33	62.67	100.00	77.05	81.33
CHB04	99.45	99.18	99.73	99.45	99.45
CHB05	96.49	97.60	95.38	96.53	96.49
CHB06	85.04	92.31	77.78	86.06	85.04
CHB07	98.89	98.41	99.36	98.88	98.89
CHB08	92.34	91.90	92.79	92.31	92.34
CHB09	100.00	100.00	100.00	100.00	100.00
CHB10	99.76	99.76	99.76	99.76	99.76
CHB11	93.89	93.32	94.46	93.86	93.89
CHB12	61.46	46.21	76.70	54.52	61.46
CHB13	70.52	81.09	59.95	73.34	70.52
CHB14	70.44	58.39	82.48	66.39	70.44
CHB15	91.93	82.93	93.33	73.52	91.93
CHB16	86.90	73.81	100.00	84.93	86.90
CHB17	98.05	97.87	98.23	98.05	98.05
CHB18	93.20	86.39	100.00	92.70	93.20
CHB19	94.69	90.27	99.12	94.44	94.69
CHB20	79.25	58.49	100.00	73.81	79.25
CHB21	95.41	91.89	98.92	95.24	95.41
CHB22	98.70	97.93	99.48	98.69	98.70
CHB23	97.61	95.98	99.25	97.57	97.61
CHB24	87.80	88.91	86.70	87.94	87.80
Mean	89.45	84.72	93.86	87.67	89.45

### 3.3 Ablation study

**FIGURE 6:**
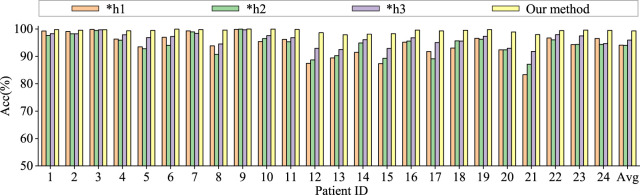
Ablation study for epilepsy recognition CHB-MIT dataset of different graph learning.∗h1 represents the use of spatial distance graph learning only. ∗h2 indicates the use of functional connectivity graph learning only. ∗h3 denotes the use of adaptive graph learning only.

**FIGURE 7:**
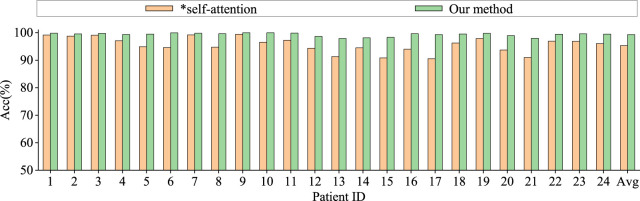
Ablation study for epilepsy recognition CHB-MIT dataset of multi-head attention.

To assess the contributions of different components in our model to classification performance, we conduct comparative experiments using model based only on similarity graph representation, model based only on distance graph representation, model based only on trainable graph representation, and model without multi-head attention. To ensure the fairness of the experiments, the model settings are kept consistent. The results are shown in Figures 6, 7.

Graph convolutional network uses only spatial distance graph learning, denoted as ∗h1. Graph convolutional network uses only functional connectivity graph learning, denoted as ∗h2. Graph convolutional network uses adaptive graph learning, denoted as ∗h3. Non-attention graph representation models refers to the model that does not employ self-attention mechanisms to obtain self-attention scores for the three graph representations. We directly concatenate the representations of three graphs into a graph feature, denoted as ∗self-attention.

From the Figures 6, 7, it is evident that MGCNA outperforms the other four ablation experiments. Compared to our model in this paper, the accuracy of the four ablation experiment models is lower by 5.22 % , 5.30 % , 3.37 % , and 3.99 % , respectively. As illustrated in Figure 6, the trainable graph representation model demonstrates superior performance compared to the other two graph representation learning methods, particularly for patients ♯ 4, ♯ 12, ♯ 13, ♯ 14, ♯ 17, and ♯ 21. Between spatial distance graph learning and functional connectivity graph learning, the latter more effectively captures the interrelationships among EEG signals.

The effect of the self-attention mechanism on epilepsy detection approximates that of the model using only distance graph representation. Figure 7 illustrates that allocating different attention weights to graph features through self-attention mechanism helps to capture dependencies between graph features and enhances the model’s ability to model relationships between graph features, which can achieve good performance in epilepsy detection. The model can effectively utilize the spatiotemporal relationships among EEG signals, supplement information through learnable graph representations, and obtain attention scores through self-attention mechanisms, and therefore the model’s learning capability has been significantly enhanced.

**FIGURE 8:**
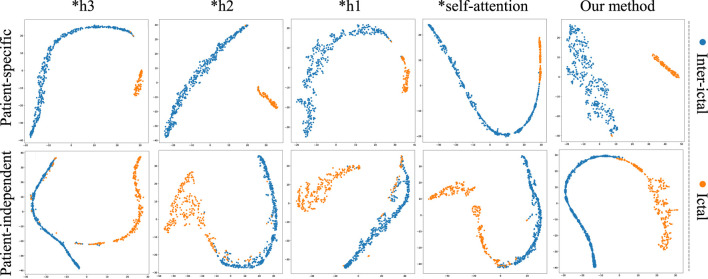
The t-SNE visualization in 2D embedding space of interictal and ictal features by comparing the models from ablation study.

To further validate the superiority of MGCNA, t-SNE is applied to visualize and analyze the features extracted from the ablation study. As depicted in Figure 8, the t-SNE visualization in two-dimensional embedding space illustrates the interictal and ictal features for both patient-specific and patient-independent experiments. We can see that in both patient-specific and patient-independent experiments, our approach exhibits superior recognition capabilities compared to the ablated models. Particularly, models utilizing only one graph construction method tend to confuse some interictal and ictal features. In contrast, better discriminative features were obtained using MGCNA, mainly in terms of significant inter-ictal distances and dense intra-ictal distributions. These observations indicate that combining multi-branch GCN with self-attention mechanisms can yield the optimal performance for epileptic seizure classification.

## 4 Discussion

### 4.1 The influence of thresholds on graph representations

In both functional connectivity graph learning and spatial distance graph learning methods, it is necessary to set a threshold for constructing an adjacency matrix. The constructed adjacency matrix must not only ensure the sparsity of the graph but also be capable of distinguishing temporal and spatial characteristics of different types of EEG data to enhance the accuracy of the model in identifying epileptic seizures.

**FIGURE 9:**
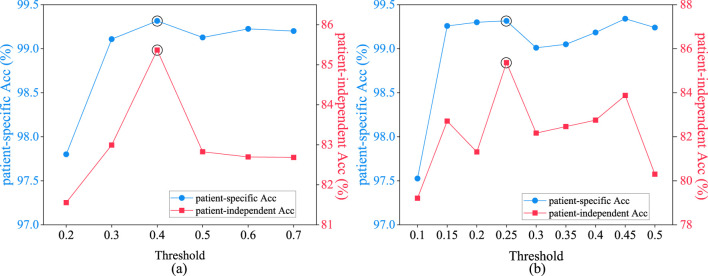
Performance comparison of patient-specific and patient-independent experiments with different thresholds. **(A)** spatial distance graph; **(B)** functional connectivity graph.

Figure 9A depicts the average epileptic seizure detection results of the CHB-MIT dataset in patient-specific and patient-independent experiments, under varying thresholds $R_{1}$ for spatial distance graph learning. The threshold range selected spans from 0.2 to 0.7. Notably, at a threshold of 0.2, the patient-specific epileptic seizure detection accuracy is the lowest, as lower thresholds tend to introduce excessive irrelevant physical connections between unrelated nodes in the spatial distance graph. Therefore, to maintain the sparsity of the adjacency matrix and achieve optimal seizure detection results, configuring the threshold $R_{1}$ for spatial distance graph learning to 0.4 is advocated.

Figure 9B illustrates the average epileptic seizure detection results in both patient-specific and patient-independent experiments under various threshold values $R_{2}$ for functional connectivity graph learning. The threshold range selected spans from 0.1 to 0.5, with a stride of 0.05. From Figure 9, it is evident that $R_{2}$ has a more pronounced impact on patient-independent epileptic seizure detection results. In the patient-specific experiment, where the training and testing datasets originate from the same patient, they exhibit similar data distributions. However, in the patient-independent experiment, differences exist between various patients, leading to inconsistent effects of different thresholds on the patients. Appropriate thresholds are applied to eliminate unrelated channel signals, utilizing functional connectivity to capture neuronal synchronized discharge during epileptic seizures (Abbas et al., 2021). To maximize the spatial discriminative power of the functional connectivity graph and achieve optimal results for seizure detection, the threshold $R_{2}$ for functional connectivity graph learning is set at 0.25.

**FIGURE 10:**
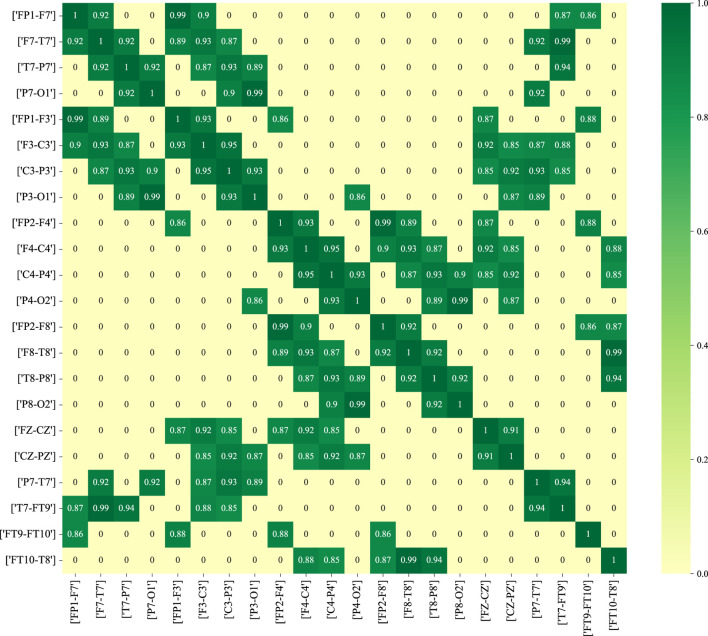
The spatial distance graph learning of EEG signals under thresholds $R_{1}$ set as 0.4.

The spatial distance graph learning based on Equation 3 with the threshold $R_{1}$ set to 0.4 is illustrated in Figure 10. The distance between two bipolar derivations was determined by measuring the separation between the centers of the bipolar derivations. For instance, the coordinates of FP1-F3 are the centers of the FP1 coordinates and the F3 coordinates. After calculating the Euclidean distances, these distances are normalized to create the distance graph representation, and self-connections are added, i.e., the graph representation of the Euclidean distances plus the diagonal matrix.

**FIGURE 11:**
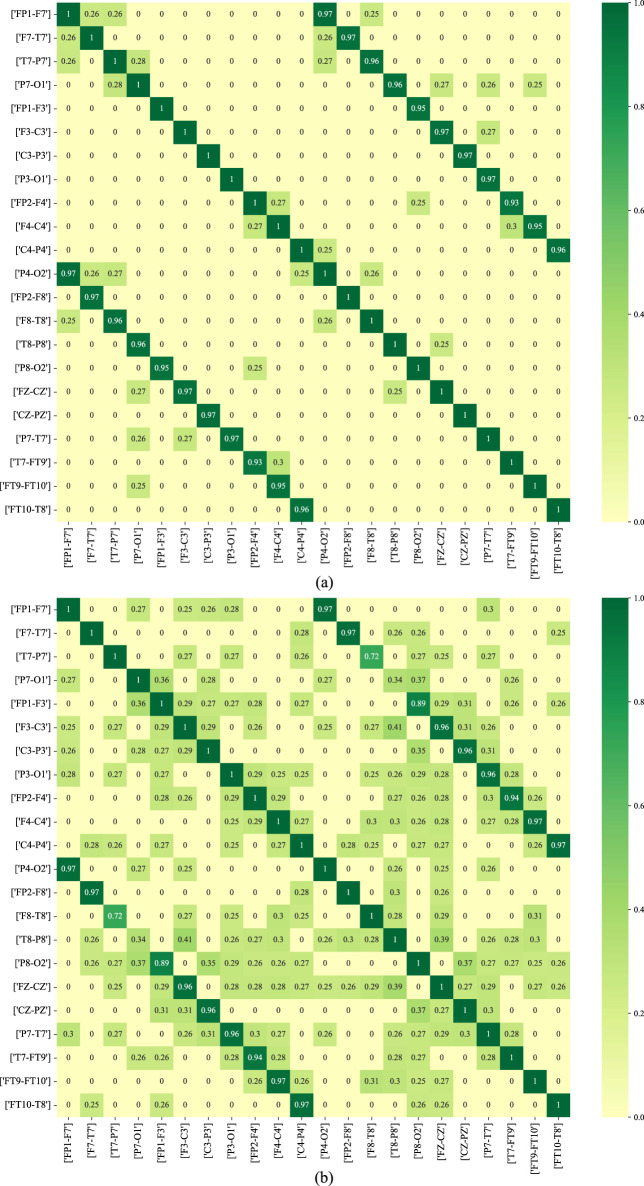
The functional connectivity graph learning of interictal and ictal signals under thresholds $R_{2}$ set as 0.25. **(A)** ictal signal; **(B)** interictal signal.

Figure 11 depicts the functional connectivity graph learning of interictal and ictal signals under thresholds set at 0.25. In the figure, we have selected and described the functional connectivity graph learning that represents interictal and ictal signals with the highest degree of similarity. The values in the graph are calculated as the Pearson correlation coefficients, which are then normalized into a similarity adjacency matrix ranging from 0 to 1. From Figure 11, it can be observed that functional connectivity graph learning for interictal signals exhibits a strong degree of similarity, while the ictal signal displays weaker inter-correlations across many bipolar derivations. Graph representations based on functional connectivity can depict the functional connections between brain regions as interdependencies among EEG signals.

### 4.2 Comparisons with state-of-the-art methods

Table 3 presents a performance comparison between the proposed MGCNA and state-of-the-art epilepsy detection algorithms in the patient-specific experimental setting. Most of these methods involve feature extraction and deep learning algorithms for epilepsy detection. It is evident that the advancement of deep learning methods holds significant importance for signal recognition and detection.

**TABLE 3:** Comparisons of performance between proposed method and recent works (patient-specific experimental setting).

Method	Acc (%)	Sens (%)	Spec (%)
Channel selection + KNN (Ein Shoka et al., 2021)	85	86.04	83.78
Multiscale spectral features + RF (Sukriti et al., 2021)	98.9	98.12	**99.17**
normalization + CNN (Cimr et al., 2023)	96.99	97.06	96.89
WNG-TS-1DCNN (Wang et al., 2023b)	93.1	91.8	96.3
HAN (Zhao et al., 2023b)	98.3	97.34	96.07
GAT + BiLSTM (He et al., 2022)	98.52	97.75	94.34
CNN + Transformer (Zhao et al., 2023a)	98.76	97.70	97.60
proposed MGCNA	**99.32**	**98.74**	98.40

The bold values represent the highest values in each column.

Machine learning methods are classical approaches for epilepsy detection. Ein Shoka et al. (2021) conducted feature extraction after channel selection and evaluated the performance of different classifiers for epilepsy detection, with KNN exhibiting the best classification performance. Sukriti et al. (Sukriti et al., 2021) extracted multiscale spectral features (MSSFs) and employed a random forest classifier for epilepsy classification exhibits a higher specificity compared to MGCNA (99.17 % *versus* 98.4 % ). However, the MGCNA achieves higher accuracy and sensitivity than that method. Cimr et al. (2023) achieved EEG classification by normalizing input signals and an 8-layer depth CNN model. The MGCNA is superior to that method in terms of accuracy, specificity and sensitivity. Zhao et al. (2023a) proposed using CNNs to extract local features and transformers to capture global information. Although MGCNA outperforms this approach, the differences in preprocessing methods also affect the results of epilepsy detection.

GCN can analyze signals by considering the three-dimensional spatial positions of EEG electrodes, thereby compensating for potential spatial information loss in deep learning methods such as CNN. The introduction of GCN can be utilized to analyze the spatiotemporal correlations among channels. Methods based on GCN have already demonstrated excellent performance for the detection of epileptic signals. Wang et al. (2023b) introduced a two-stream graph-based framework for learning the Weighted Neighbour Graph (WNG) representation in both the frequency and time domains. However, this approach achieved inferior accuracy, sensitivity, and specificity, with values of 93.1 % , 91.8 % , and 96.3 % , respectively, in comparison to the method proposed in the current study. Zhao et al. (2023b) introduced a hybrid Attention Network that utilizes the GAT to extract spatial features and the Transformer to extract temporal features addresses the issue of imbalanced data. However, in the patient-specific experiment, it achieved slightly lower accuracy, specificity, and sensitivity compared to MGCNA. He et al. (2022) utilized the GAT to extract spatial features and employed a BiLSTM network to capture temporal features, achieving an accuracy of 98.52 % on the CHB-MIT dataset, slightly lower than MGCNA.

Table 4 presents comparisons of performance between the proposed method and state-of-the-art epilepsy detection algorithms in the patient-independent experimental setting. Due to the substantial variations in signals among different patients, the overall performance of the patient-independent experiment is lower than that of the patient-specific experiment. The accuracy of the patient-independent experiment based on SVM (Shoeb, 2009) only reaches 58.32 % , indicating a significant performance gap compared to MGCNA. Wei et al. (2019) proposed an epilepsy detection algorithm by combining CNN and Wasserstein Generative Adversarial Nets (WGANs), achieving lower sensitivity than MGCNA, i.e. 72.11 % vs. 83.54 % , respectively. Zhao et al. (2023b) introduced the HAN model, which performs epilepsy detection not only in patient-specific experiments but also in patient-independent experiments. In both types of experiments, the three performance metrics were lower than MGCNA. Additionally, the ablation studies conducted in the patient-independent experimental setting are showcased herein, demonstrating that the MGCNA method exhibits superior performance compared to each ablation study. Since the three graph learning models can better extract the structural information of EEG and perform graph feature fusion, the overall performance is better than other epilepsy detection algorithms.

**TABLE 4:** Comparisons of performance between proposed method and recent works (patient-independent experimental setting).

Method	Acc (%)	Sens (%)	Spec (%)
SVM (Shoeb, 2009)	58.32	74.6	42.19
WGANs (Wei et al., 2019)	84.00	72.11	**95.89**
HAN (Zhao et al., 2023b)	73.15	72.75	75.7
∗h1	82.49	80.33	85.74
∗h2	85.21	79.83	91.27
∗h3	84.72	80.83	89.16
∗self-attention	84.19	79.49	89.56
proposed MGCNA	**85.45**	**84.72**	93.86

The bold values represent the highest values in each column.

Through the above analysis, the MGCNA framework offers several key advantages. First, it employs a multi-branch graph convolutional network structure that dynamically learns temporal correlations and spatial topological information, enhancing the ability to process complex EEG signals, particularly in capturing spatial and temporal dependencies in epileptic brains. Second, the use of three graph learning approaches allows for a comprehensive evaluation of connectivity and synchronization across multiple channels, improving adaptability in different patient scenarios. Additionally, the multi-head attention mechanism further strengthens the framework’s ability to handle local features and complex EEG patterns. Experimental results demonstrate that MGCNA outperforms other methods in both patient-specific and patient-independent tasks, highlighting its strong generalization capabilities. Lastly, as an end-to-end automatic seizure detection model, MGCNA can be applied in clinical decision-making, helping clinicians diagnose childhood epileptic seizures more quickly and accurately, providing significant practical value.

### 4.3 Limitations and future work

While the MGCNA achieved satisfactory results in both patient-specific and patient-independent experiments, the MGCNA has several limitations. Firstly, spatial distance graph learning requires the prior determination of channel locations and is not robust to variations in EEG channel count. If channels are missing or if channel locations change, it can adversely affect recognition performance. Secondly, our model has a relatively long computation time because it involves calculating Pearson correlation coefficients for each sample to extract features for constructing EEG graph representations. Finally, the performance of model may depend on the quality and diversity of the data it has been trained on, and it is crucial to validate it on larger, more diverse datasets to ensure its generalizability. Specifically, both functional connectivity graph learning and spatial distance graph learning employ thresholds.

In future work, it is essential to explore graph representation methods that are more suitable for epileptic signals. While many scholars have already employed various graph representations (Raeisi et al., 2022), these often involve manual feature engineering and have long computation times. In the future, it’s possible to explore alternative composition methods or utilize clustering algorithms to identify the most suitable adjacency matrix. In the future, we will explore alternative graph generation techniques, such as imposing appropriate constraints on trainable adjacency matrix or using clustering algorithms to identify the most suitable graph generator. Identifying the most suitable graph representation method for epileptic signal recognition is of paramount importance. In this study, patient-independent experiments hold more clinical relevance, and there is significant room for improvement in accuracy. To capture common seizure characteristics among different patients, techniques such as transfer learning will be considered to enhance the accuracy of patient-independent experiments. In current research, considerable attention has been devoted to the occurrence of seizures, yet there exist variations in seizure types among individuals. Consequently, in future investigations, emphasis will be placed on the analysis of seizure types in epilepsy research.

## 5 Conclusion

This study proposes a children epilepsy detection model named as MGCNA that combines a multi-branch GCN with multi-head attention. The MGCNA leverages three graph structures to learn spatiotemporal features among channels. It uses spatial graph representations to capture spatial distances between channels, functional connections between channels to learn spatial dependencies in the signals, and employs learnable graph representations to complement spatiotemporal features. The model employs a multi-head attention to assign importance weights to graph signals, learning relationships between graph representations. The model’s performance in classifying epileptic EEG signals is validated on the CHB-MIT dataset through patient-specific and patient-independent experiments. The experimental results indicate that the MGCNA shows excellent performance of childhood seizure detection surpassing other existing methods. This method can be used to assist in the childhood seizure detection and effectively reduce the workload of physicians. The EEG classification algorithm introduced in this research provides the potential to establish an EEG monitoring system for children with epilepsy.

## Data Availability

The original contributions presented in the study are included in the article/supplementary material, further inquiries can be directed to the corresponding authors.
